# Gene expression and proliferation biomarkers for antidepressant treatment resistance

**DOI:** 10.1038/tp.2017.16

**Published:** 2017-03-14

**Authors:** J Breitfeld, C Scholl, M Steffens, G Laje, J C Stingl

**Affiliations:** 1 Research Division, Federal Institute for Drugs and Medical Devices (BfArM), Bonn, Germany; 2 Washington Behavioral Medicine Associates, LLC, Chevy Chase, MD, USA; 3 Centre for Translational Medicine, University Bonn Medical Faculty, Bonn, Germany

## Abstract

The neurotrophic hypothesis of depression suggests an association between effects on neuroplasticity and clinical response to antidepressant drug therapy. We studied individual variability in antidepressant drug effects on cell proliferation in lymphoblastoid cell lines (LCLs) from *n*=25 therapy-resistant patients versus *n*=25 first-line therapy responders from the Sequenced Treatment Alternatives to Relieve Depression (STAR*D) study. Furthermore, the variability in gene expression of genes associated with cell proliferation was analyzed for tentative candidate genes for prediction of individual LCL donor's treatment response. Cell proliferation was quantified by EdU (5-ethynyl-2′-deoxyuridine) assays after 21-day incubation of LCLs with fluoxetine (0.5 ng μl^−1^) and citalopram (0.3 ng μl^−1^) as developed and described earlier. Gene expression of a panel of candidate genes derived from genome-wide expression analyses of antidepressant effects on cell proliferation of LCLs from the Munich Antidepressant Response Signature (MARS) study was analyzed by real-time PCR. Significant differences in *in vitro* cell proliferation effects were detected between the group of LCLs from first-line therapy responders and LCLs from treatment-resistant patients. Gene expression analysis of the candidate gene panel revealed and confirmed influence of the candidate genes ABCB1, FZD7 and WNT2B on antidepressant drug resistance. The potential of these genes as tentative biomarkers for antidepressant drug resistance was confirmed. *In vitro* cell proliferation testing may serve as functional biomarker for individual neuroplasticity effects of antidepressants.

## Introduction

Depressive disorders are among the leading causes of disability worldwide^1^ with a lifetime prevalence of more than 16%.^2^ Depression contributes to decreased quality of life including morbidity, loss of productivity and suicidal thoughts.^3^

The neurotrophic hypothesis of depression suggests a chronic hyperactivity of the hypothalamic–pituitary–adrenal axis leading to a lowered growth factor expression and trophic changes in the brain.^4^ The hippocampus, a cerebral structure involved in emotion processing and stress response seems to be the most affected area in depression-associated neurotrophic changes.^5^ A recent meta-analysis considering 8927 samples from 15 different magnetic resonance imaging studies confirmed significant lower hippocampal volumes in depressed patients compared with healthy controls.^6^ Antidepressant treatment has been shown to be associated with reversing hippocampal atrophy by the enhancement of neuronal proliferation and synaptic plasticity.^7^ Furthermore, treatment efficacy in patients with smaller hippocampus volume has been observed to be delayed over the time of 3–4 weeks,^8^ which is usually the earliest time for reliable evaluation of treatment efficacy.^9^ This phenomenon, together with the usually low (<30%) response rates to first-line antidepressant medications,^10, 11^ point to a need to identify response or non-response biomarkers for the prediction of individual antidepressant treatment effects.

Here, we want to identify possible functional biomarkers on neuroplasticity effects of antidepressants for associations with treatment response and resistance in patient-derived lymphoblastoid cell lines (LCLs) from the Sequenced Treatment Alternatives to Relieve Depression (STAR*D) study. LCLs are emerging tools in the field of personalized medicine research to study individual drug effects *ex vivo* (for example, reviewed in refs 12, 13). In addition, recently identified tentative gene expression biomarkers for neuroplasticity in antidepressant drug response will be studied for confirmation in this independent cohort from the STAR*D study: In a genome-wide approach using patient-derived LCLs from the Munich Antidepressant Response Signature (MARS) study, we identified five potential gene expression biomarkers that have been associated with cell proliferative effects of antidepressants (*ex vivo*) or with LCL donor's clinical response/remission in antidepressant drug therapy: transcription factor 7-like 2 (*TCF7L2*), frizzled class receptor 7 (*FZD7*), wingless-type MMTV integration site family member 2B (*WNT2B*), p-glycoprotein (*P-GP, ABCB1*) and sulfotransferase 4A1 (*SULT4A1*).

## Materials and methods

### Cell lines and study population

Lymphoblastoid cell lines from the STAR*D project were purchased from the NIMH Center for Collaborative Genetic Studies, Rodgers repository (Bethesda, MD, USA). The STAR*D study (ClinicalTrials.gov Identifier: NCT00021528) is an open label, randomized, multicenter, controlled clinical study that aimed to study effective subsequent treatment strategies after a first unsuccessful antidepressant therapy.^14^ The study consisted of four treatment levels. After each level, responders were allocated to a 12-month follow-up period during which the patients were further monitored and treated with the beneficial treatment regimen. Patients who experienced non-response or intolerable side effects entered the subsequent treatment level.

All patients were first-line treated with a citalopram monotherapy at the first level of this study. In level 2, they had to choose between adding another antidepressant (bupropion or buspirone) and switching to different medication (with random assignment to sertraline, bupropion or venlafaxine). In a similar way, level 3 consisted of either add-on (lithium or triiodothyronine) or switch (mirtazapine or nortriptyline). During the final level, all previous medications were taken off and patients were randomly assigned to one of two treatment strategies: monotherapy with tranylcypromine versus combination of venlafaxine and mirtazapine.

A total of 50 cell lines were obtained (Table 1), derived from *n*=25 responders to the first level (citalopram) of treatment and from *n*=25 treatment-resistant patients who did not show response after undergoing the whole treatment algorithm (level 4). All patients were patients of Caucasian origin, for first-line treatment (level 1) all have been treated with citalopram monotherapy in defined doses ranging from 5 to 40 mg. Depressive symptoms were rated by Quick Inventory of Depressive Symptomatology (QIDS)^15^ over the course of up to 14 weeks. The complete clinical data are listed in the Supplementary Information. LCLs were ordered to cover *n*=25 first-line-responders (with more than 50% decline of depressive symptoms during the first month), and *n*=25 treatment-resistant patients (with no response or remission during the whole treatment algorithm of the STAR*D study). No group differences for age, sex or illness severity between responders and treatment-resistant patients was detected. The participating patients gave informed consent to provide biomaterial for the study of antidepressant response biomarkers also including generation of cell lines.

### Cell culture and proliferation assays

LCLs were cultured in RPMI medium and incubated with antidepressants for 21 days as described elsewhere.^16^ Fluoxetine and citalopram (Sigma-Aldrich, Taufkirchen, Germany) stock solutions were prepared in dimethyl sulfoxide. Proliferation rates were measured using the EdU (5-ethynyl-2′-deoxyuridine) incorporation assays (Thermo Fisher Scientific, Darmstadt, Germany; catalog no. C10635) as reported before.^16^ Relative proliferation rates were calculated as ratio between treated and untreated samples of the same cell lines.

### Nucleic acid extraction and gene expression analysis

RNA was extracted using the NucleoSpin RNA Kit (Machery-Nagel, Düren, Germany; catalog no. 740955). Preparation of cDNA and RT-PCR experiments were carried out as already published.^16^ QuantiTect and custom-made primers were purchased from Qiagen (Hilden, Germany) and Eurofins Genomics (Ebersberg, Germany), respectively (Table 2) These primers allow the measurement of gene expression levels of five candidate biomarker genes (Table 2) that have been recently identified by whole genome gene expression experiments of LCLs derived from depressed patients participating in the MARS study.^16^ The basal gene expression was indicated as ?CT values, gene expression fold changes were calculated by ΔΔCT method using GAPDH as reference gene.^16, 17, 18^

### Statistical analyses

To test for differences between antidepressant-treated and -untreated proliferation rates in the same cell lines, the paired *t*-test was used. Between the groups of responders and treatment-resistant patients, the proliferation rates were compared with Student's *t*-tests. To measure the strength of the relationship between the proliferation rates of citalopram- and fluoxetine-incubated cells, Spearman's correlation coefficient (*ρ*) was calculated. In dependence of the nature of the data types of the clinical covariates (gender, anxiety status, menopausal status), either parametric (Student's *t*-tests, Spearman correlation) or nonparametric tests (Wilcoxon–Mann–Whitney rank-sum test, Spearman's rank correlation) were used when analyzed with respect to the proliferation rates and gene expression data. As expression levels between various treatment conditions and cell lines were largely different, nonparametric tests (Wilcoxon–Mann–Whitney rank-sum test) were given preference to check for statistically significant group differences concerning fold changes after the *in vitro* treatment with antidepressants. The statistical power amounts to 93.4% for EdU phenotyping experiments and to 99.9% for RT-PCR validation experiments with effect sizes of *r*=2 and significant levels of *α*=0.05 each. All the *P*-values are reported as nominal *P*-values and are unadjusted for multiple testing. The statistical analyses were carried out using IBM SPSS Statistics 21 (Ehningen, Germany).

## Results

### Phenotyping of LCL proliferation via EdU-incorporation assays

**Figure 1:**
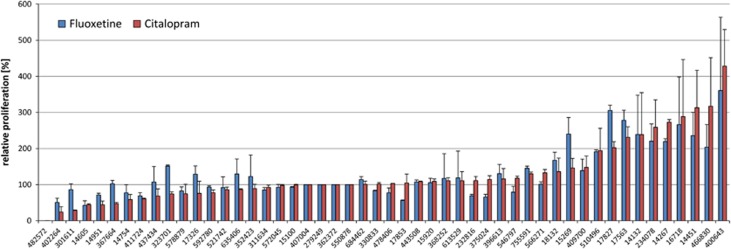
Results from EdU phenotyping experiment. The relative LCL proliferation rates show large individual differences among the cell lines. EdU, 5-ethynyl-2′-deoxyuridine; LCL, lymphoblastoid cell line.

**Figure 2:**
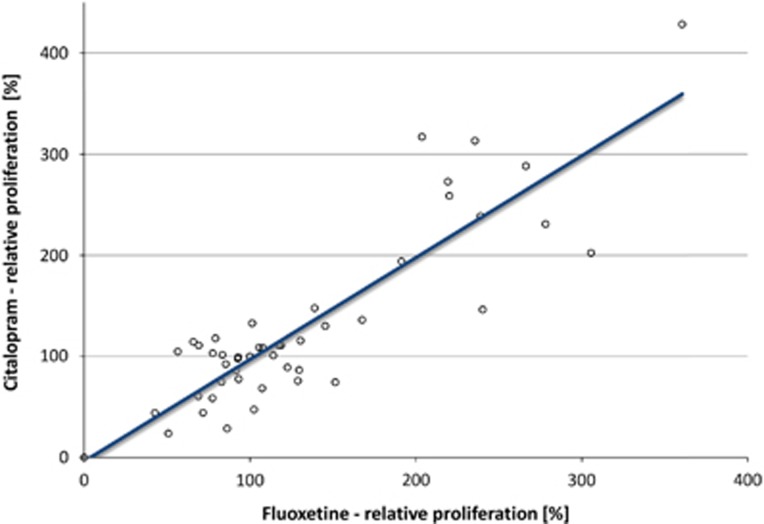
Correlation plot of relative LCL proliferation rates after *in vitro* treatment of LCLs for 3 weeks with fluoxetine (*x* axis) and citalopram (*y* axis). A significant correlation was detected (*ρ*=0.875). LCL, lymphoblastoid cell line.

**Figure 3:**
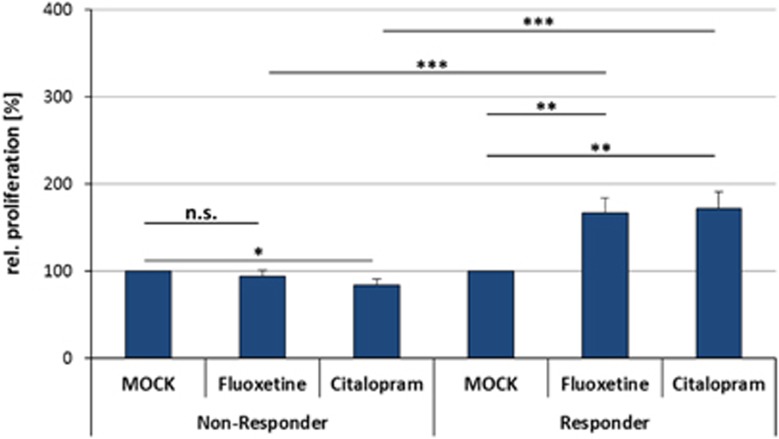
Relative LCL proliferation rates of non-responder- and responder-derived cell lines treated with fluoxetine or citalopram for 21 days or mock-treated control samples from the same cell lines. LCL proliferation rates were significantly increased in responder-derived cell lines and decreased in non-responder-derived cell lines treated with citalopram. Significant differences between responder- and non-responder-derived cell lines were observable (deviations are indicated as standard error; *P<0.05, **P<0.01, ***P<0.001). LCL, lymphoblastoid cell line.

**Figure 4:**
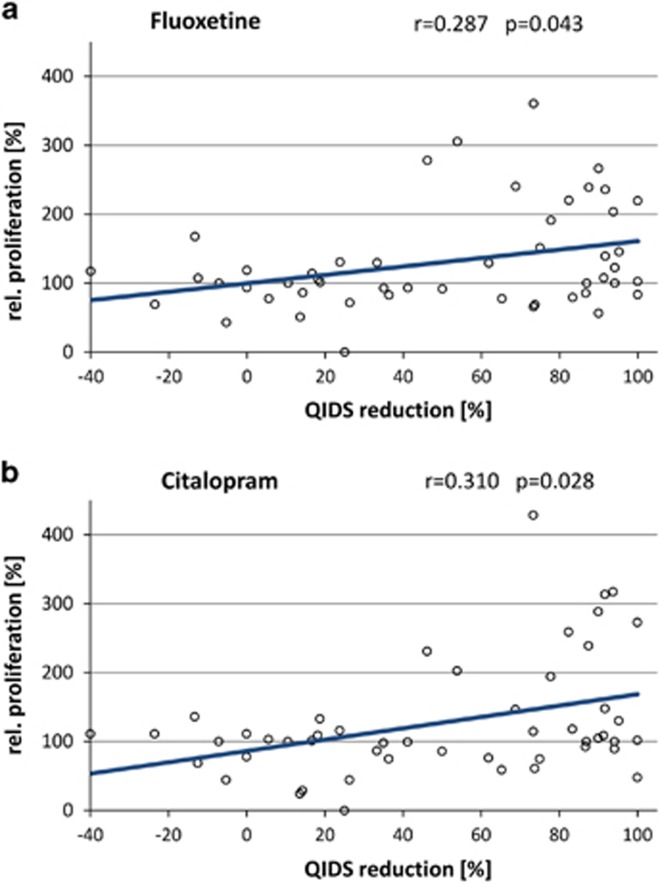
Correlation plots of patients' QIDS reduction and relative proliferation after *in vitro* treatment with (**a**) fluoxetine or (**b**) citalopram. QIDS, Quick Inventory of Depressive Symptomatology.

After incubation of the LCLs with therapeutical concentrations of citalopram (0.3 ng μl^−1^) or fluoxetine (0.5 ng μl^−1^) for 3 weeks, EdU-based proliferation phenotyping experiments revealed strong interindividual differences between single cell lines (Figure 1). The relative LCL proliferation rates ranged from 0.0 to 428.4%. Averaged over all 50 cell lines, the overall proliferative effects were reported compared with MOCK-treated controls (set to 100%): fluoxetine mean 130.34%±56.32 (*P*=0.006) and citalopram mean 127.59%±61.00 (*P*=0.026). A significant correlation between the fluoxetine- and citalopram-mediated (both selective serotonin reuptake inhibitors (SSRIs)) LCL proliferation rates was detected (*ρ*=0.875, *P*<0.001; Figure 2). The differences in relative LCL proliferation rates between the two groups of first-line responders versus treatment-resistant patients were investigated. The effects in cell lines from the first-line responding patients showed significantly increased LCL proliferation after *in vitro* treatment with fluoxetine and citalopram, whereas the cell lines derived from treatment-resistant patients showed low and even decreased LCL proliferation after incubation with fluoxetine and citalopram, respectively (Figure 3). A positive correlation between percentage QIDS reduction and LCL proliferation was detected by Spearman's correlation analysis for both citalopram- (*ρ*=0.310, *P*=0.028) and fluoxetine-treated (*ρ*=0.287, *P*=0.043) cell lines (Figure 4). The covariates analyses revealed no significant associations between LCL proliferation and either gender (*P*_Fluoxetine_=0.142, *P*_Citalopram=_0.052), age (*ρ*_Fluoxetine_=−0.802, *P*_Fluoxetine_=0,581; *ρ*_Citalopram_=0.054, *P*_Citalopram_=0.710), menopausal status (*P*_Fluoxetine_=0.731, *P*_Citalopram_=0.416) or anxiety status (anxious versus non-anxious depression; *P*_Fluoxetine_=0.771, *P*_Citalopram_=0.330).

### Gene expression analyses of the candidate genes

Previously derived tentative gene expression biomarkers were further investigated within this STAR*D cohort: Three of the tested candidate genes (*FZD7*, *TCF7L2* and *WNT2B*) are substantial part of the canonical Wnt signaling pathway, which has a key role in the regulation of neurogenesis and synaptic plasticity. The transporter and drug metabolism enzyme genes *ABCB1* and *SULT4A1* are involved in neuroprotection and metabolism of neuroactive substances, respectively.

**Figure 5:**
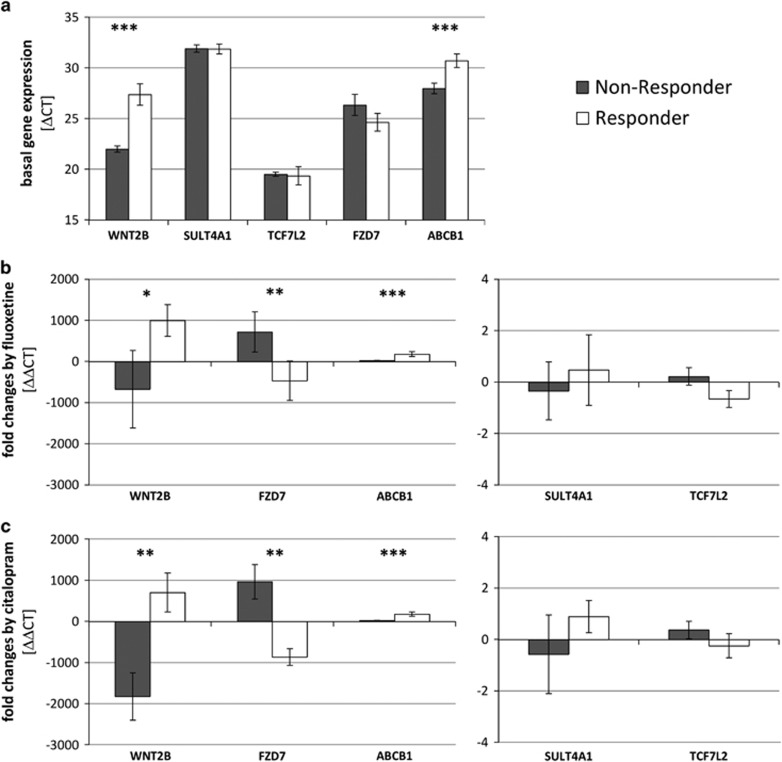
Results of gene expression experiments of the candidate genes. (**a**) Basal gene expression indicated as difference of maximal cycle number and ?CP values of untreated samples. Gene expression fold changes after 21-day *in vitro* treatment of LCLs with fluoxetine (**b**) or citalopram (**c**) (deviations are indicated as standard error; *P<0.05, **P<0.01, ***P<0.001). LCL, lymphoblastoid cell line.

Within the STAR*D LCL cohort, the gene expression of those five genes was measured—including basal gene expression and gene expression after 3 weeks of *in vitro* incubation with therapeutical concentrations of fluoxetine (0.5 ng μl^−1^) and citalopram (0.3 ng ml^−1^). The association of gene expression changes and LCL donor's clinical status was investigated. Significant differences between the responder-derived cell lines and the cell lines derived from treatment-resistant patients in basal gene expression of *WNT2B* (*P*=0.0001) and *ABCB1* (*P*=0.009) were detected. Previous experiments using LCLs from the MARS study showed no associations of clinical parameters with the basal gene expression of these gene.^16^ No significant differences were found for genes *FZD7* (*P*=0.643), *TCF7L2* (*P*=0.355) or *SULT4A1* (*P*=0.943; Figure 5a), which is in accordance with previous results except for gene *SULT4A1* (in the MARS study, *FZD7* and *TCF7L2* genes only showed correlations between fluoxetine-induced fold changes and cell proliferation).^16^ The basal gene expression of *SULT4A1* was low and only detectable in 11 out of 50 cell lines (*n*=5 non-responder-derived cell lines versus *n*=6 responder-derived cells). The fold changes of *WNT2B* (FC_Fluoxetine_Responder_=995.17 and FC_Fluoxetine_Non-Responder_=−675.69 with *P*_Fluoxetine_=0.046, FC_Citalopram_Responder_=701.78 and FC_Citalopram_Non-Responder_=−1828.48 with *P*_Citalopram_=0.003), *FZD7* (FC_Fluoxetine_Responder_=−469.20 and FC_Fluoxetine_Non-Responder_=720.03 with *P*_Fluoxetine_=0.003, FC_Citalopram_Responder_=−869.02 and FC_Citalopram_Non-Responder_=963.93 with *P*_Citalopram_=0.002) and *ABCB1* (FC_Fluoxetine_Responder_=175.05 and FC_Fluoxetine_Non-Responder_=18.30 with *P*_Fluoxetine_=0.009, FC_Citalopram_Responder_=174.48 and FC_Citalopram_Non-Responder_=20.64 with *P*_Citalopram_=0.010) after *in vitro* treatment with fluoxetine and citalopram show significant associations with LCL donor's clinical therapy resistance status (Figures 5b and c). These fold changes represent temporal changes after treatment of LCLs with fluoxetine and citalopram. In previous experiments from the MARS cohort, only associations of *WNT2B* fold changes with LCL donor's clinical remission were reported. No significant associations of gene expression fold changes of *TCF7L2* (FC_Fluoxetine_Responder_=−0.66 and FC_Fluoxetine_Non-Responder_=0.21 with *P*_Fluoxetine_=0.140, FC_Citalopram_Responder_=−0.24 and FC_Citalopram_Non-Responder_=0.37 with *P*_Citalopram_=0.369) and *SULT4A1* (FC_Fluoxetine_Responder_=0.47 and FC_Fluoxetine_Non-Responder_=−0.35 with *P*_Fluoxetine_=0.548, FC_Citalopram_Responder_=0.89 and FC_Citalopram_Non-Responder_=−0.58 with *P*_Citalopram_=0.413) with the LCL donor's clinical treatment resistance were found in this recent study. However, highly significant correlations were found between proliferation rates and the gene expression fold changes of ABCB1 (*P*<0.001), FZD7 (*P*=0.009) and WNT2B (*P*<0.001) in both the treatment groups.

## Discussion

### LCL cell proliferation rate as antidepressant response marker

The proliferation rates of 50 LCLs derived from depressed patients were determined after 3 weeks of incubation with therapeutical concentrations of the antidepressant drugs fluoxetine and citalopram. Cell proliferative effects were significantly stronger in the *n*=25 cell lines derived from the group of first-line reponder patients from the STAR*D study compared with the group of *n*=25 treatment-resistant patients. These findings support the neurotrophic hypothesis of antidepressant's action, which suggests an antidepressant-mediated reversal of impaired hippocampal structure and activity.^19^ The induction of neural proliferation is linked to an enhanced neuroplasticity, which in turn, leads to a normalization of the depressed brain function.^7^ This explanation helps to understand the delay in symptomatic improvement because cerebral remodeling processes are complex and time-consuming. Such proliferative effects of antidepressants were frequently reported, for example, in human neuronal precursor cells derived from embryonic stem cells^20^ or in hippocampal granule cells of adult mice^21^ (both after incubation with fluoxetine). Another study found—after 25 days of treatment with imipramine—an increased hippocampal synaptogenesis and neurogenesis in a rat model of depression.^22^ The biological mechanisms behind these phenomena are still poorly understood. Thus, it has been reported that neurotrophic factors obtain a key role in antidepressant's action,^23^ and that antidepressant effects are restricted to type 2 but not type 1 neuronal progenitor cells accelerating the maturation of neurons.^24, 25^ Fluoxetine might convey the integration of newborn neurons into the particular functional networks (for example, in the dentate gyrus network or the hippocampal pyramidal cells of the hypothalamic–pituitary–adrenal axis) leading to an improved cellular survival.^26^ The individual proliferative effects observed here after long-term incubation with antidepressant drugs do not correlate with *in vivo* proliferative effects. Another study using LCLs evaluated sensitivity to antidepressants as growth inhibition by the SSRI paroxetine.^27^ Antidepressant drug effects seem to show a high interindividual variability resulting in such opposing effects. However, the concentrations used were tenfold higher than the concentrations used to assess proliferative stimuli. Also the differences in the time of onset of growth inhibition (24 or 48 h) versus proliferative effects (3 weeks) were very different. No signs of cell proliferation stimulus on blood cells or bone marrow have ever been described for antidepressant drug therapy, but this *ex vivo* effect in the cell lines of depressed patients may contribute to the puzzle of explaining the high variability in antidepressant efficacy observed in clinical routine. Thus, even though the applicability of peripheral proliferation after long-term incubation with antidepressants as response biomarker seems limited, investigation of molecular backgrounds and the identification of potential gene expression biomarkers associated with neuro-proliferation or -protection might be advantageous.

### Gene expression of candidate genes

The most notable difference in expression levels between responder- and treatment resistance-derived LCLs were observed for *WNT2B*, *FZD7* and *ABCB1*. We found significant elevated basal gene expression levels of the genes *WNT2B* (?CT difference of 4.96) and *ABCB1* (?CT difference of 2.31) in the LCLs derived from patients with antidepressant treatment resistance relative to responder-derived ones. Further, in responder-derived cell lines, fold changes by SSRIs were significantly increased for the genes *WNT2B* (up to 2530-fold higher), *FZD7* (up to 1833-fold higher) and *ABCB1* (up to 157-fold higher). ABCB1 is the best studied member of the ABC transporter superfamily possessing a key role in cellular detoxification and transmembrane transport across the blood–brain barrier. The allocrite spectrum is broad and includes neurotoxic agents (for example, glucocorticoids, drugs and xenobiotics) and thus, ABCB1 holds neuroprotective effects eventually resulting in an increased response to antidepressants. Peripheral glucocorticoids are stress response factors in the hypothalamic–pituitary–adrenal axis, and they normally have toxic effects on neurons and are suspected to be causative for depressions.^28^ Many antidepressants such as amitriptyline, citalopram, doxepin, fluoxetine or paroxetine are substrates for transport by Pgp at the blood–brain barrier influencing brain bioavailability of central nervous system (CNS) active drugs, and overexpression of Pgp has been described to be associated with treatment resistance to various antidepressant drugs.^29^ Carriers of defined haplotypes within the *ABCB1* gene show decreased risk of developing depressions,^30^ and polymorphisms of the *ABCB1* gene are thought to predict adverse antidepressant drug effects^31^ indicating a role of *ABCB1* in depression. Further, a high expression of *ABCB1* in the blood–brain barrier (resulting from chronical antidepressant treatment) might account for an increased clearance of neurotoxic agents (for example, glucocorticoids) from the brain. Peripheral glucocorticoids are stress factors of the HPA axis, are toxic to neurons and suspected to be risk factors for depressive disorders. These ABCB1-mediated neuroprotective effects could contribute to an increased proliferation of neuronal cells and to a modulation of neuroplasticity.

*WNT2B* and *FZD7* are elements of the canonical WNT signaling pathway regulating neurogenesis, synaptic plasticity and dendritic arborization.^32^ Downstream growth factors such as FGF, BDNF and BMP are involved in depression pathogenesis as well as in the maintenance of adult neurogenesis.^33, 34, 35^ WNT2B is a highly conserved signal peptide and a ligand for members of the frizzled transmembrane receptor family; FZD7 belongs to this family of GPCRs. Wnt glycoproteins usually are liberated from astrocytes and show short-ranged action. An activated Wnt signaling pathway controls stem cell pluripotency and tissue regeneration,^36^ and regulates the expansion of CNS progenitor cells.^37^ Furthermore, WNT proteins support the differentiation of specific glial neuronal precursors,^38^ and are involved in immunological processes of microglia^39^—macrophage-like cells of the brain that are required for CNS homeostatic functions.^40^ Neurotoxic agents reduce *WNT* expression in developmental hippocampal neurons^41^ and an impaired hippocampal Wnt signaling is associated with a decreased neurogenesis, and an increase of depression-like behavior in adult rats.^42^ Wnt signaling is stimulated by antidepressive drugs,^43^ and however, no effects are recognizable in constitutively activated pathways^44^ indicating a role of Wnt signaling in antidepressant's action. In accordance to these findings, we reported elevated gene expression levels of WNT2B and lowered gene expression levels of FZD7 after *in vitro* incubation with SSRIs in responder-derived LCLs. As FZD7 inhibits the WNT signaling while WNT2B enhances it, chronic antidepressant treatment strongly activates this pathway. This results in an increased stem cell liberation and differentiation to neurons (neurogenesis). Further, this leads to an improved maintenance of adult hippocampal neurogenesis, expansion of CNS progenitor cells, as well as CNS development in general. The detailed molecular mechanisms are not understood so far but it is assumed that these effects take place by integration of newborn type 2 neurons into neuronal networks. All these effects together might be responsible for an enhanced neuronal plasticity and achieving remission from depression.

## Conclusions

Peripheral proliferation of LCLs derived from depressed patients with known response status revealed significant differences between cell lines derived from treatment-resistant patients and from responders. Furthermore, general proliferative effects of both fluoxetine and citalopram were detected supporting the neurotrophic hypothesis of antidepressant's action. The low response rates as well as the high variability in antidepressant efficacy observed in clinical practice could be based on individual proliferative effects within the depressed brain and an individual susceptibility to antidepressant-mediated changes in neuroplasticity. Furthermore, candidate gene expression biomarkers that have been previously identified using MARS LCLs and are associated with neuro-proliferation or -protection were evaluated. This was supported by the fact that SSRI-mediated gene expression fold changes of *WNT2B*, *FZD7* and *ABCB1* were correlated with proliferation rates. Significant differences between LCLs derived from responders and treatment-resistant patients for these genes were confirmed rendering them as potential temporal mediators or baseline predictors, which eventually advance the personalized treatment approach in depressions in the future.

## Figures and Tables

**Table 1:** Characteristics of the STAR*D LCL study cohort

	*Responder*	*Treatment-resistant patients*
*Gender*		
Male	*n*=10	*n*=14
Female	*n*=15	*n*=11
		
Age	48.12±13.8	48.96±9.5
*QIDS*		
Week 0	17.3±3.2	18.5±3.1
Week 14	2.6±1.9	15.5±3.9

Abbreviations: LCL, lymphoblastoid cell line; QIDS, Quick Inventory of Depressive Symptomatology; STAR*D, Sequenced Treatment Alternatives to Relieve Depression.

**Table 2:** Primers used for real-time PCR experiments

*Gene*	*Assay name or sequence*	*Recent findings in MARS LCLs*
*WNT2B*	Hs_WNT2B_va.1_SG	Association of fold changes by fluoxetine with LCL donor's clinical remission
*SULT4A1*	Hs_SULT4A1_1_SG	Association of basal gene expression with LCL donor's clinical response
*ABCB1*	Hs_ABCB1_1_SG	Association of basal gene expression with LCL proliferation
*TCF7L2*	Hs_TCF7L2_1_SG	Association of basal gene expression and fold changes by fluoxetine with LCL proliferation
*FZD7*	Fwd: 5′-CCTTCCCCTTCTCATGCCC-3′ Rev: 5′-CAGCCCGACAGGAAGATGAT-3′	Association of fold changes by fluoxetine with LCL proliferation
*GAPDH*	Hs_CACNA2D3_1_SG	Housekeeping gene

Abbreviations: LCL, lymphoblastoid cell line; MARS, Munich Antidepressant Response Signature.
