# Supplementary material for: Gene expression and proliferation biomarkers for antidepressant treatment resistance
Source: Transl Psychiatry. 2017 Mar 14;7(3):e1061–. doi: 10.1038/tp.2017.16 (PMC5416664; doi:10.1038/tp.2017.16)
Supplement: Supplementary Information [file tp201716x1.docx]

**Supplement**

**Supplementary Table 1: Clinical data of patient derived LCLs participating in the STAR*D study.**
